# Pneumococcic Meningitis and Meningismus

**Published:** 1914-07

**Authors:** 


**Pneumococcic Meningitis and Meningismus.** Huber, in Archives of Pediatrics, May, 1914, reports four cases of pneumonia with well-marked cerebral symptoms with coma, the cerebrospinal fluid being free from bacteria.—C. G. L-W.

